# Toll-like receptor 3: a double-edged sword

**DOI:** 10.1186/s40364-025-00739-5

**Published:** 2025-02-23

**Authors:** Marvin L Hsieh, Daisuke Nishizaki, Jacob J Adashek, Shumei Kato, Razelle Kurzrock

**Affiliations:** 1 Medical College of Wisconsin, Milwaukee, WI USA; 2 Moores Cancer Center, University of California San Diego, La Jolla, CA USA; 3 Department of Oncology, The Sidney Kimmel Comprehensive Cancer Center, The Johns Hopkins Hospital, Baltimore, MD USA; 4 MCW Cancer Center and Genomic Sciences and Precision Medicine Center, Medical College of Wisconsin, Milwaukee, WI USA

**Keywords:** Toll-like receptor 3 (TLR3), TLR3 agonist, Cancer, Immunotherapy, Viral infection, Autoimmune disease, Allergy

## Abstract

The discovery of Toll-like receptors (TLRs) and their role in dendritic cells earned the Nobel Prize for 2011 because TLRs profoundly enhanced our understanding of the immune system. Specifically, TLR3 is located within the endosomal compartments of dendritic cells and plays a crucial role in the immune response by acting as a pattern recognition receptor that detects both exogenous (viral) and endogenous (mammalian) double-stranded RNA. However, TLR3 activation is a double-edged sword in various immune-mediated diseases. On one hand, it can enhance anti-viral defenses and promote pathogen clearance, contributing to host protection. On the other hand, excessive or dysregulated TLR3 signaling can lead to chronic inflammation and tissue damage, exacerbating conditions such as autoimmune diseases, chronic viral infections, and cancer. In cancer, TLR3 expression has been linked to both favorable and poor prognoses, though the underlying mechanisms remain unclear. Recent clinical and preclinical advances have explored the use of TLR3 agonists in cancer immunotherapy, attempting to capitalize on their potential to enhance anti-tumor responses. The dual role of TLR3 highlights its complexity as a therapeutic target, necessitating careful modulation to maximize its protective effects while minimizing potential pathological consequences. In this review, we explore the intricate roles of TLR3 in immune responses across different disease contexts, including cancer, infections, autoimmune disorders, and allergies, highlighting both its protective and detrimental effects in these disorders, as well as progress in developing TLR3 agonists as part of the immunotherapy landscape.

## Background

Toll-like receptors (TLRs), for which Ralph Steinman, Jules Hoffmann, and Bruce Beutler were awarded the Nobel Prize in 2011, revolutionized the understanding of human innate immunity and anti-microbial defense [1, 2]. TLRs have since emerged as key components of the innate immune system that play a fundamental role in detecting pathogen-associated molecular patterns (PAMPs) and activating both innate and adaptive immune responses [3]. These receptors are essential in recognizing a broad range of pathogens, including viruses, bacteria, fungi, and parasites, initiating immune responses that are vital for host defense.

As pattern recognition receptors, TLRs can induce pro-inflammatory and anti-viral responses upon recognizing specific PAMPs or danger-associated molecular patterns that are typically found in infectious agents or damaged cells. In humans, 10 types of TLRs form a cohesive defense barrier against a broad spectrum of pathogens, with each TLR being specifically activated by distinct PAMPs and triggering unique signaling pathways to promote pathogen clearance [4]. TLR3, for example, is primarily activated by viral double-stranded RNA (dsRNA), while other TLRs recognize a variety of microbial components: TLR1/2 detect bacterial lipoproteins, TLR4 senses lipopolysaccharide (LPS) from Gram-negative bacteria, and TLR5 is activated by bacterial flagellin. Each TLR activates specific signaling pathways that ultimately lead to the production of cytokines, chemokines, and interferons (IFNs), which are critical for pathogen clearance and initiating immune responses.

The TLR network is central to many immune processes, including anti-viral, cancer, and auto-immunity [5]. In particular, TLR3 has gained attention for its versatile role in these diverse biological contexts, offering a rich terrain for understanding the complexity of host defense mechanisms and disease pathogenesis. TLR3 serves as a cellular sensor for dsRNA, which initiates downstream signaling cascades that augment inflammation and cell death [6]. A crucial downstream molecule in this pathway is type I IFN (IFN-α and IFN-β), which plays a central role in the antiviral immune response. Upon TLR3 activation, IFNs induce an antiviral state in neighboring cells, inhibiting viral replication and enhancing immune cell clearance of infected cells, crucial for effective antiviral defense and immune homeostasis. Thus, the production of IFNs is vital for orchestrating an effective antiviral defense and maintaining immune homeostasis during viral infections. The IFN-driven immunostimulatory effect is critical to understanding the multifactorial role TLR3 plays across various immunological pathways.

The immunological significance of TLR3 has encouraged the development of dsRNA analogues as TLR3 agonists to exploit the therapeutic potential of this pro-inflammatory pathway. Despite some success in early clinical trials, the definitive efficacy of TLR3 agonists in both monotherapy and combinatorial regimens is yet to be established [7]. This review aims to delve into the specific mechanisms through which TLR3 influences distinct immune pathways and the biological implications for optimizing the effectiveness of TLR3 agonists.

## Biology of TLR3

**Fig. 1:**
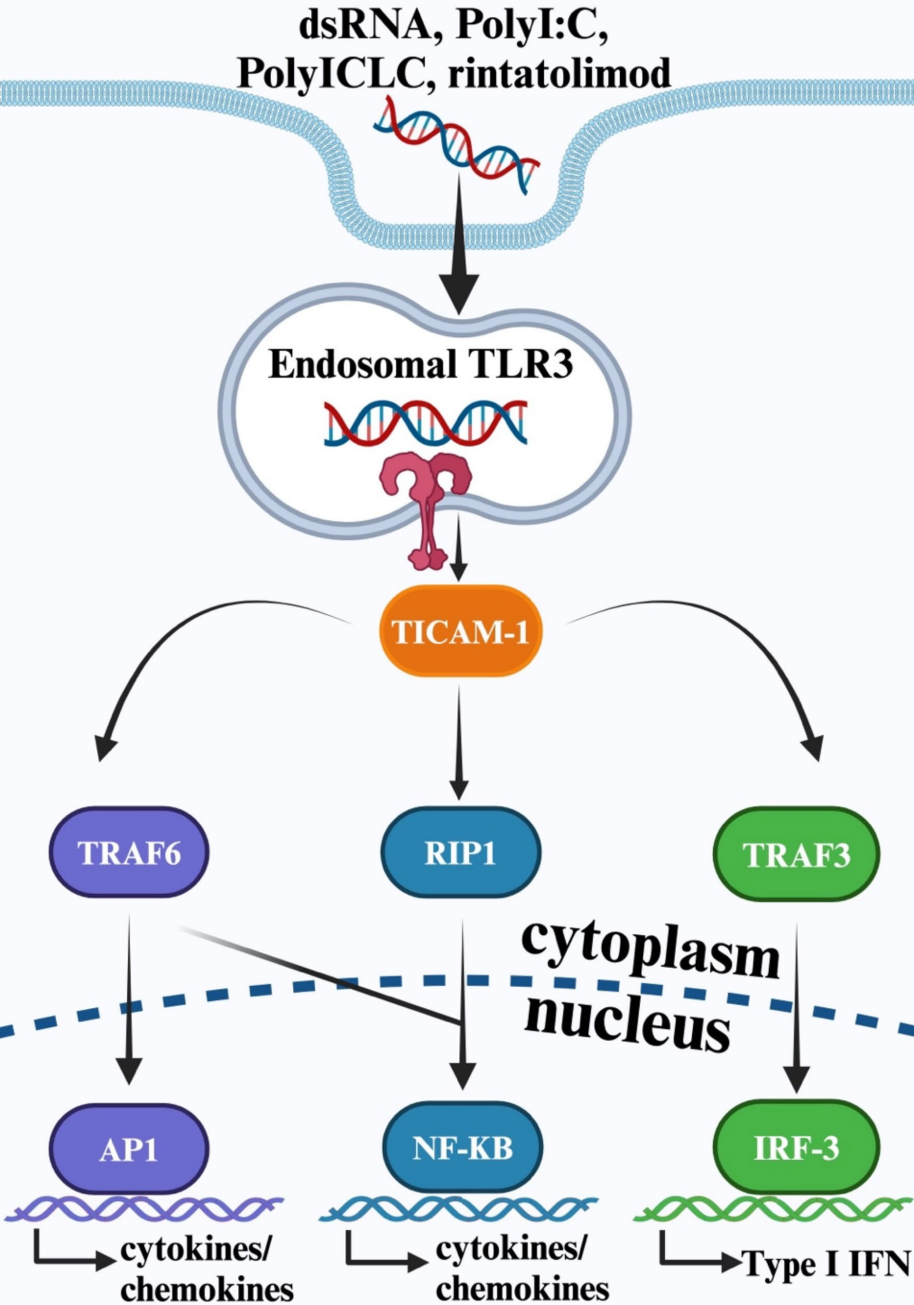
TLR3 signaling pathway. Binding of dsRNA to endosomal TLR3 activates TICAM-1 complex, which induces downstream upregulation of transcription factors in the nucleus. Activation of AP-1, IRF-3, and NF-κB promotes production of type I IFNs and inflammatory cytokines. Created by BioRender Abbreviations: AP1 = Activator protein 1; dsRNA = double-stranded ribonucleic acid; IRF-3 = Interferon regulatory factor 3; NF-κB = Nuclear factor kappa B; Poly I: C = Polyinosinic: polycytidylic acid; Poly ICLC = Polyinosinic-Polycytidylic Acid Stabilized with Polylysine and Carboxymethylcellulose; RIP1 = Receptor interacting protein 1; TICAM-1 = Toll/interleukin-1 receptor domain-containing adapter molecule-1; TLR3 = Toll-like receptor 3; TRAF3 = Tumor necrosis factor receptor-associated factor 3; TRAF6 = Tumor necrosis factor receptor-associated factor 6.

TLR3 plays a critical role in the innate immune response by recognizing and responding to dsRNA, a common hallmark of viral infections [6]. In humans, TLR3 is predominantly expressed within the immune system and localized to the endosomal compartments of myeloid dendritic cells (DCs), where it can detect internalized dsRNA from viral pathogens [8–10] (Fig. 1). Notably, structural studies have shown that while TLR3 binds dsRNA as short as 46 base pairs, efficient activation requires longer RNA strands, which facilitate cooperative binding and clustering of TLR3 dimers along the dsRNA helix for robust signal transduction [11, 12]. However, TLR3 is also expressed in non-immune cells, including epithelial cells, fibroblasts, and endothelial cells, which provides an efficient barrier to pathogens [13]. TLR3’s presence in non-immune cells broadens its functional scope, bridging innate immunity and tissue-specific responses to enhance immunity in pathogen-exposed tissues.

Upon recognition of dsRNA, TLR3 triggers a signaling cascade that involves the activation of Toll/interleukin-1 receptor domain-containing adapter molecule-1 (TICAM-1) [14]. TICAM-1 serves as a crucial adaptor protein that subsequently activates key transcription factors, such as Activator Protein 1 (AP-1), interferon regulatory factor 3 (IRF-3), and nuclear factor kappa B (NF-κB). These factors collaborate to induce the expression of type I IFNs and other inflammatory cytokines, creating an environment that is unfavorable for viral replication [14, 15]. Notably, TLR3 activation by dsRNA has been demonstrated to upregulate the expression of anti-viral genes in CD8 + dendritic cells, emphasizing its crucial role in the induction of anti-viral gene expression during CD8 + T cell responses [15, 16].

It is important to note that while TLRs is evolutionarily conserved in many vertebrates, including humans and mice, significant species-specific differences in expression and regulation exist [17]. In humans, TLR3 is primarily expressed in dendritic cells, whereas in mice, it is predominantly found in macrophages and is strongly induced by lipopolysaccharide (LPS)—a response not observed in humans [18]. These differences can be attributed to variations in the non-coding regions and promoter sequences of the TLR3 gene, leading to distinct expression patterns between species. Since murine models are often the standard system for studying infections and immune modulation, these species-specific differences should be considered a limitation when extrapolating findings from mouse models to human immune responses.

## Role of TLR3 in Human diseases

**Fig. 2:**
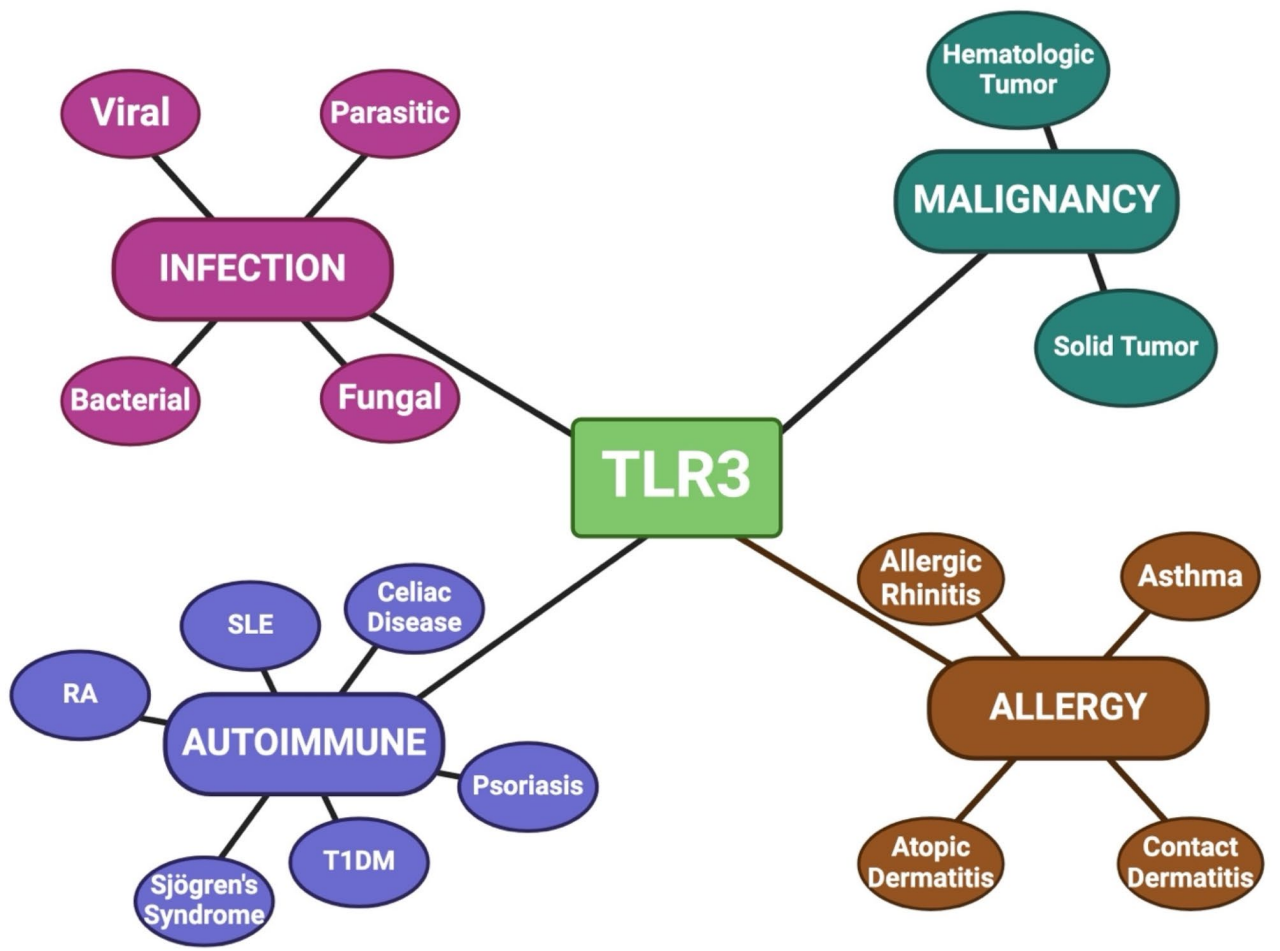
TLR3 plays a multifactorial role in the pathogenesis of infections, cancer, autoimmune disease, and allergy. Created by BioRender Abbreviations: RA = Rheumatoid Arthritis; SLE = Systemic Lupus Erythematosus; T1DM = Type 1 Diabetes Mellitus; TLR3 = Toll-like receptor 3

As a potent stimulator of both the innate and adaptive immune defenses, TLR3 closely regulates the pathogenesis and outcomes in human immunopathology. TLR3 activation promotes a pro-inflammatory state systemically and locally, serving as a mediator for both protective and detrimental effects across infectious diseases, cancer, autoimmune diseases, and allergy (Fig. 2).

### Cancer

TLR3 has multiple roles in cancer. For instance, TLR3 is likely associated with both tumor stimulatory and inhibitory effects. TLR3 agonists are in clinical trials for patients with malignancies, though efficacy has not yet been shown.

#### TLR3 expression can correlate with both better and worse prognoses in cancer patients

Both good and poor prognoses have been observed in TLR3 research, suggesting that the clinical implications of TLR3 in oncology should not be taken with a one-size-fit-all approach [7]. Increased TLR3 expression is associated with better survival outcomes in hepatocellular carcinoma, melanoma, non-small cell lung carcinoma (NSCLC), neuroblastoma, and colorectal carcinoma [19–24]. In contrast, a worse prognosis was observed with increased TLR3 expression in oral squamous cell carcinoma and prostate cancer [25, 26]. Both good and poor prognoses have been reported in esophageal, gastric, breast, and renal cell carcinoma [25, 27–35].

The protective role of TLR3 against tumorigenesis may be driven by TLR3 activation of the pro-apoptotic pathway and intercellular immune interactions [36, 37]. For instance, in NSCLC cell lines, TLR3 not only induces cell death through caspase activation, but also enhances the anti-tumor response through CD103 + DCs antigen presentation [38]. Polyinosinic: polycytidylic acid (Poly I: C) treatment in tumor cells activates the CD103 + cell subpopulation, which functions to direct CD8 T-cell response through uptake and presentation of apoptotic cell antigens [39]. Activation of TLR3 is necessary for coordinating the pro-apoptotic response mediated by CD103 + DCs, highlighting the pivotal role of TLR3 in coordinating a multifaceted anti-tumor immune response. Furthermore, TLR3 activation through Poly I: C induces macrophage polarization towards a more tumor suppressive state in acute myeloid leukemia [40]. TLR3 agonism increases levels of CD11c + M1 macrophages, which are associated with increased phagocytosis and the consequent pro-inflammatory, anti-tumor immunity. These findings underscore the significance of TLR3 in orchestrating immune responses that collectively contribute to a more favorable cancer prognosis.

The pro-tumor capabilities of TLR3 activation are not as well-characterized as their anti-tumor counterparts; however, several mechanisms have been proposed in TLR3-associated tumorigenesis leading to recurrence and metastasis. TLR3 activation with Poly I: C can induce breast cancer cells to express phenotypes of cancer stem cells (CSCs) that possess tumorigenic properties [41]. This mechanism acts simultaneously through NF-κB and β-catenin signaling pathways, promoting resistance to chemotherapeutic drugs, increased colony-forming capacities, and tumor cell aggregation. The generation and dissemination of CSCs has been proposed as the basis of metastases, which may explain worsened clinical outcomes associated with TLR3 expression in some cancer subtypes [42]. The pro-inflammatory microenvironment promoted by TLR3 activation of NF-κB may also be a prognostic risk factor in cancer progression, as observed in many cancers, such as head and neck squamous cell carcinoma, colon carcinoma, and hepatocellular carcinoma [43–45]. NF-κB has long been recognized as a tumorigenic marker, driving inflammation that not only accelerates error-prone DNA replication in tumor cells but also shields them from apoptosis during tumor progression [46]. Beyond these effects, NF-κB contributes to tumorigenesis through additional mechanisms, including generating DNA damage via reactive species, enhancing the transcription of genes that promote cancer cell migration and invasion, and stimulating angiogenesis [47]. Considering TLR3 signaling and its downstream modulators have the capacity to amplify known hallmarks of cancer through mechanisms such as NF-κB activation, DNA damage via reactive species, enhanced transcription of genes promoting invasion and angiogenesis, and the generation of a pro-inflammatory microenvironment, its role in tumorigenesis cannot be overlooked. While TLR3 activation can stimulate acute inflammation that potentially enhances anti-tumor immunity by activating dendritic cells and effector T-cells, chronic or dysregulated inflammation, a hallmark of many cancers, fosters an immunosuppressive tumor microenvironment that supports tumor progression, metastasis, and therapy resistance [48, 49]. This duality underscores the need for careful consideration and monitoring of TLR3 agonists in anti-tumor therapies, as they may shift the inflammatory balance to either drive tumor regression or inadvertently promote tumorigenesis depending on the context and duration of treatment.

#### Current immunotherapies with TLR3 in Cancer

Immunotherapy has transformed cancer outcomes [50–54]. The growing understanding of TLR3 activation in anti-tumor pathways has spurred investigations into the clinical potential of TLR3 agonists as anti-neoplastic agents (Table 1). TLR3, through its recognition of viral dsRNA, triggers a robust immune response via type I IFN production, activation of NK cells, and induction of apoptosis. This pathway has been harnessed in preclinical studies showing that co-cultures with the TLR3 agonist Poly I: C can directly induce apoptosis in various cancer cell types, including breast, cervical, melanoma, colorectal, hepatocellular carcinoma, oral squamous cell carcinoma, and lung cancer [7, 55]. These findings underscore the potential of TLR3 as an effective target in cancer immunotherapy by stimulating both innate and adaptive immune responses to combat tumor cells.

**Table 1:** Current status of development of TLR3 agonists *

Drug Name	Trade Name	Company	Mechanism of Action	Number of clinical trials registered in NCT (clinicaltrials.gov)	Eligible Diseases
Rintatolimod	Ampligen	AIM ImmunoTech	Restricted TLR3 Agonist	**25** 11: Completed 1: Not yet recruiting 3: Recruiting 4: Suspended 4: Terminated 2: Withdrawn	Cancer: *N* = 17 Viral: *N* = 8
Poly ICLC	Hiltonol	Oncovir	TLR3 Agonist	**131** 15: Active, not recruiting 59: Completed 4: Not yet recruiting 25: Recruiting 20: Terminated 6: Withdrawn 2: Unknown	Allergy: 1 Cancer: 122 Viral: 5 Healthy: 3

*(Data search as of April 26th, 2024)

Abbreviations: NCT = National Clinical Trial; Poly ICLC = Polyinosinic-Polycytidylic Acid Stabilized with Polylysine and Carboxymethylcellulose; TLR3 = Toll-like receptor 3

In order to reduce the toxicity and enhance the stability of TLR3 agonists in human therapy, three Poly I: C derivatives were introduced clinically: polyadenylic: polyuridylic acid (poly A: U), rintatolimod (Ampligen), poly ICLC [56, 57]. These TLR3 agonists have been evaluated in clinical trials, demonstrating varying efficacy in both monotherapies and combinatorial regimens (Table 2) [58–65]. The TLR3-mediated immune activation by these derivatives is believed to enhance the anti-tumor effects through both direct tumor cell death and the potentiation of immune system activation.

**Table 2:** Examples of clinical trials in cancer evaluating TLR3 agonists with efficacy

Drug	Regimen	Phase	Cancer type	Results	NCT number	Ref.
**Poly A: U**	Poly A: U only vs. saline	N/A	Operable breast cancer	5-year OS = 82% vs. 72% 5-year RFS = 72% vs. 56%	N/A	[59]
	Poly A: U with 5-FU and doxorubicin vs. 5-FU and doxorubicin	III	Locally advanced gastric cancer	5-year OS = 68.4% vs. 52.4% 10-year OS = 55.6% vs. 43.8% 15-year OS = 50.1% vs. 38.1% 5-year RFS = 68.3% vs. 52.1% 10-year RFS = 60.3% vs. 46.6% 15-year RFS = 59.4% vs. 44.1%	N/A	[61]
**Rintatolimod (Ampligen)**	αDC1 Vaccine + Celecoxib, Interferon Alfa-2b, Rintatolimod	I/II	Peritoneal surface malignancies	Median PFS for colon cancer: - moderately differentiated = 20.5 months - poorly differentiated = 8.9 months Median PFS for appendiceal cancer: - Low-grade = 50.4 months - Intermediate-grade = 34.2 months - High-grade = 8.9 months	NCT02151448	[62]
	Celecoxib, Interferon Alfa-2b, Rintatolimod	II	Colorectal cancer metastatic to the liver	ORR = 0% Median PFS = 1.5 months (90% CI 1.4–1.8) Median OS = 10.5 months (90% CI 2.2–15.2)	NCT03403634	[63]
**Poly ICLC (Hiltonolol)**	Poly ICLC only	II	Recurrent or progressive anaplastic glioma	ORR = 11.1% (95% CI, 0.04–0.24) 24% of patients had not progressed at 6 months Median OS = 43 weeks	NCT00058123	[58]
	Poly ICLC only	II	Pediatric low-grade gliomas	70% of patients had not progressed at 6 months 6-month OS = 100% 2-year OS = 100%	NCT01188096	[64]
	Poly ICLC only	II	Glioblastoma multiforme	6-month PFS = 30% 12-month survival rate = 69%	NCT00052715	[60]
	Poly ICLC with antigen vaccine	I/II	Glioblastoma multiforme	ORR = 14.3% (SL-701 + Poly ICLC + bevacizumab) ORR = 2.2% (SL-701 + GM-CSF + Imiquimod) 12-month OS = 42.9% (SL-701 + Poly ICLC + bevacizumab) 12-month OS = 17.4% (SL-701 + GM-CSF + Imiquimod) 6-month PFS = 26.7% (SL-701 + Poly ICLC + bevacizumab) 6-month PFS = 46.6% (SL-701 + GM-CSF + Imiquimod)	NCT02078648	[65]

**Abbreviations**: αDC1 = alpha-type-1 polarized dendritic cell; 5-FU = 5-Fluorouracil; GM-CSF = granulocyte-macrophage colony-stimulating factor; N/A = Not applicable; NCT = National Clinical Trial; ORR = objective response rate; OS = overall survival; PFS = progression free survival; Poly A: U = Polyadenylic–Polyuridylic Acid; Poly ICLC = Polyinosinic-Polycytidylic Acid Stabilized with Polylysine and Carboxymethylcellulose; RFS = Recurrence-Free Survival; SL-701 = Subcutaneously Injected Multivalent Glioma-Associated Antigen Vaccine; TLR3 = Toll-like receptor 3

Poly A: U was first evaluated in a French clinical trial in 1980 as an adjuvant treatment for operable breast cancer [59]. Monotherapy with Poly A: U showed activity and safety in a randomized trial, as patients in the treatment arm benefited from higher overall survival and relapse-free survival after 5 years compared to the standard-of-care arm (surgery +/- radiotherapy). These findings were echoed in a Korean phase III trial in the 1980s that assessed the same molecule’s capabilities in chemo-immunotherapy for locally advanced gastric cancer after curative surgery [61]. Administered in combination with 5-fluorouracil and doxorubicin, poly A: U prolonged overall survival and prevented recurrence in patients receiving the drug regimen. No other clinical trials since have evaluated poly A: U in cancer patients. This is likely due to its limited standalone efficacy without combination therapy and its potential to enhance tumorigenic pathways in TLR3-expressing tumor cells [66, 67]. Nevertheless, the development of safer and more potent analogues has carried the torch in exploring TLR3 agonism as an anti-tumor therapy.

Rintatolimod, known for its ability to induce cell maturation and promote interleukin-12 (IL-12) production in DC’s, has shown promise in immunotherapy [68]. While no ongoing trials utilize rintatolimod as a monotherapy, the drug’s safety and optimal dosage as an adjuvant for a HER-2/neu peptide vaccine is being evaluated in a randomized phase I/II breast cancer trial (NCT01355393). Chemokine modulation therapy, which is composed of rintatolimod, IFN-α2b, and celecoxib, has been evaluated as a treatment regimen (NCT03403634) and as an adjuvant for a dendritic cell vaccine (NCT02151448), pembrolizumab (NCT03599453, NCT05756166), and paclitaxel (NCT04081389). These trials aim to exploit the ability of rintatolimod to enhance immune activation and promote a more robust anti-tumor response via TLR3 signaling.

Poly ICLC, a stable and potent derivative of Poly I: C, which acts as a TLR3 agonist, is extensively researched as an adjuvant in cancer immunotherapy clinical trials. While there is currently no publicly available data suggesting that monotherapy with Poly ICLC is an effective treatment for cancer, findings from a phase II trial in recurrent anaplastic glioma patients revealed that Poly ICLC was well tolerated but did not demonstrate improvement in 6-month progression-free survival. In contrast, ongoing trials, such as a Phase II study (NCT02423863), have shown that Poly ICLC stimulates both local and systemic immune responses in patients with head and neck squamous cell carcinoma and melanoma. This trial has expanded to assess Poly ICLC as an adjuvant to immune checkpoint inhibitors, emphasizing the potential synergy between TLR3 agonism and checkpoint blockade in enhancing immune responses against tumors [69].

TLR3 agonists hold great promise in cancer immunotherapy, but their dual role in cancer pathogenesis complicates the safety and efficacy of systemic administration. The immunostimulatory effects of TLR3 agonists can result in nonspecific activation of the TLR3 pathway, causing systemic inflammation and unwanted side effects. Additionally, the endosomal localization of TLR3 presents a delivery challenge, as the large size and negative charge of these agonists hinder their ability to reach the target site effectively. As research progresses, newer TLR3 agonists are being developed to overcome the limitations of earlier molecules. For instance, TL-532, a novel agent that combines blocks of poly(I: C) and poly(A: U), has demonstrated bioavailability following parenteral injection and a favorable toxicological profile [70]. In preclinical studies, it stimulates the production of important chemokines and interleukins, without inducing systemic inflammation. Notably, TL-532 has shown the ability to reduce tumor growth in bladder cancer models and restore the response to immunogenic chemotherapy in fibrosarcoma. Another emerging strategy to enhance the therapeutic potential of TLR3 agonists is the use of nanoparticle delivery systems. This approach aims to improve the precision of TLR3-targeted therapies while minimizing off-target effects. Preclinical in vitro and animal studies have shown that nanoparticle-encapsulated Riboxxol, a dsRNA analogue, enhances immunogenicity, prolongs survival, and reduces adverse effects compared to conventional administration [71, 72]. Further research is needed to determine whether nanoparticle-based approaches can effectively optimize the therapeutic potential of TLR3 agonists while mitigating their associated challenges.

While the landscape of TLR3 immunotherapies has not yet yielded definitive successes, ongoing trials may reveal more about the precise role of TLR3 activation in clinical settings. The mechanism of TLR3-mediated immune activation—through the induction of apoptosis, immune cell activation, and cytokine production—underpins the rationale for its use in cancer immunotherapy. As more clinical trial data become available, these findings will provide critical insights into the integration of TLR3 agonists in future cancer therapies.

## Role of TLR3 in non-malignant diseases

While TLR3 can influence tumor progression through inflammation and immune modulation, its function is not limited to malignant diseases. The same pathways that mediate TLR3’s effects in the tumor microenvironment—particularly its ability to detect dsRNA and activate pro-inflammatory responses—also play crucial roles in non-malignant diseases, including infectious autoimmune disorders, and allergy.

### Role of TLR3 in Infectious diseases

The duality of TLR3 seen in cancer is also evident in the pathogenesis of infectious diseases, as evidence suggest that the dsRNA sensing capabilities of TLR3 can either attenuate or exacerbate symptoms of viral infection [73]. The contradictory effects of TLR3 expression in viral pathogenesis have been illustrated in both cellular and animal models (Table 3) [74–101]. The protective effect of TLR3 activation during infection has been shown in multiple viral groups with TLR3-deficient mice. In the absence of a functional TLR3 pathway, infected mice exhibit increased vulnerability to viral infection, elevated viral loads both systemically and in infected tissue, and exacerbated pathologic symptoms and mortality rates. TLR3 promotes its anti-viral capabilities through TICAM-1-dependent induction of type I IFNs, which may explain the enhanced survivability of wild-type (WT) infected mice compared to their TLR3 deficient counter parts [80, 88, 89]. Type I IFNs produced during viral infection are potent stimulators of both the innate and adaptive immune systems, allowing for increased recruitment and efficacy of both myeloid and lymphoid cells [102]. The activation of these immune modulators facilitates the restriction and clearance of viral infections, reinforcing the irreplaceable role of TLR3 pathway in antiviral defense.

**Table 3:** Effect of TLR3 expression in viral pathogenesis

Effects on Viral Infection	Test Subject	Intervention	Viruses	References
Protective	Mice	TLR3 Agonist	Chikungunya, Ebola, HBV, HSV-1, SARS-CoV-2	[74–79]
Protective	Mice	TLR3 Knockout	Chikungunya, CVB3, CVB4, EMCV, HSV-1, HSV-2, Polio, Rotavirus, WNV,	[80–91]
Protective	Monkey	TLR3 Agonist	Ebola	[76]
Protective	Human Cells	TLR3 Knockout	EMCV, hPIV-1, HSV-1, VSV	[92]
Protective	Human Cells	TLR3 Agonist	Chikungunya	[93]
Detrimental	Mice	TLR3 Agonist	RSV	[94]
Detrimental	Mice	TLR3 Knockout	CVB4, Influenza A, PTV, Rhinovirus, Vaccinia, WNV	[95–100]
Detrimental	Human Cells	TLR3 Agonist	RSV	[101]

Abbreviations: CVB3 = Coxsackievirus B3; CVB4 = Coxsackievirus B4; EMCV = Encephalomyocarditis Virus; HBV = Hepatitis B Virus; hPIV-1 = Human Parainfluenza Virus 1; HSV-1 = Herpes Simplex Virus 1; HSV-2 = Herpes Simplex Virus 2; PTV = Punta Toro Virus; RSV = Respiratory Syncytial Virus; SARS-CoV-2 = Severe acute respiratory syndrome coronavirus 2; TLR3 = Toll-like receptor 3; VSV = Vesicular Stomatitis Virus; WNV = West Nile Virus

Conversely, the detrimental effect of the TLR3 pathway on viral susceptibility and pathogenesis is closely related to the upregulation of immunogenic markers during infections. When compared to their TLR3-deficient counterparts upon viral infection, WT mice showed an increase in inflammatory markers, increased local viral load, and reduced survival [96, 97, 99]. In particular, infections with Punta Toro virus (PTV), influenza A, and vaccinia all led to significant increases in interleukin-6 (IL-6) in WT mice both systemically and at the infected tissues, suggesting the cytokine as a key regulator of TLR3-induced pathogenesis in viral infections. The exacerbated clinical outcomes may be attributed to the pro-inflammatory nature of IL-6, which is consistent with findings in human patients with chronic viral infections [103–106]. The ability of TLR3 to induce IL-6 release is well documented in in vitro studies, which may explain the contradictory role of TLR3 in inducing systemic infectious symptoms [107]. Interestingly, increased recruitment of CD8 + T-cells to the lungs was a common feature in TLR3-deficient mice infected with either influenza A or vaccinia. This suggests a potential mechanism for TLR3 induced pathogenesis, as CD8 + T-cell accumulation in airways has previously been associated with increase severity of lung injury in viral infections among infants [108]. The TLR3 pathway presents as a double-edged sword in viral defense. Although its activation can impede viral spread through cytotoxic and pro-inflammatory pathways, it also poses a threat by potentially exacerbating tissue damage and facilitating viral replication.

While TLR3 induction by dsRNA is a hallmark of anti-viral defense, TLR3 has also been shown to mediate inflammation in non-viral infectious diseases. Instead of an infectious origin for dsRNA, bacterial, fungal, and parasitic infections can trigger necrotic cells to release dsRNA, which subsequently upregulates inflammation through the TLR3 pathway, promoting synthesis of cytokines and IFNs [109–113]. Across the broader immunopathologic landscape of infectious diseases, TLR3 is cemented as a critical immune modulator and is essential to understanding the multifaceted nature of host defense.

### Role of TLR3 in autoimmune diseases

While TLR3’s role in cancer demonstrates its potential for both beneficial and harmful effects, similar complexities arise in autoimmune diseases, where TLR3 activation can lead to exacerbated inflammation. Increased expression and activation of TLR3 are seen in both organ-specific and systemic autoimmune diseases. Increased TLR3 mRNA in local tissues is common in autoimmune diseases such as rheumatoid arthritis (RA) and lupus nephritis, wherein increased cytokine production and immune cell recruitment are observed in the inflamed regions [114–117]. Interestingly, TLR3 activation does not induce B cell activation or produce anti-DNA antibodies, suggesting that TLR3-mediated lupus nephritis progresses through a B-cell independent mechanism.

TLR3-induced interferon production serves as a common mechanism underlying Type 1 diabetes mellitus (T1DM) and psoriasis. In T1DM, TLR3 signaling in pancreatic tissue upregulates NF-kB and IRF3, leading to extensive type I IFN production that contributes to autoimmune damage by inducing IFN-β production in beta cells via the TICAM-1-dependent pathway, triggering apoptosis and inhibiting insulin production [118–120]. Similarly, in psoriasis, dsRNA-induced activation of TLR3 in keratinocytes induces IFN-β mRNA expression, promoting dendritic cell activation and maturation [121].

In Sjögren’s syndrome (SS), induction of IFN-β and IL-6 through TLR3 agonism is necessary for accelerated disease progression in mouse salivary gland tissue and human salivary gland epithelial cells [122–124]. TLR3 is a potent stimulator of innate and adaptive immune systems, as its signaling pathway increases secretion of B-cell activating factor, a key modulator in SS pathogenesis [125]. TLR3 activation in SS also contributes to apoptosis by induction of pro-apoptotic mediators in salivary gland epithelial cells [126]. These findings may support the role of TLR3 in the pathogenesis of SS, as reduced cellular clearance of apoptotic debris has been suggested as a potential cause of inflammation in patients [127].

Celiac disease is closely regulated by TLR3 signaling, as downstream induction of interferons and cytokines is responsible for the pathogenesis in mice. TLR3 activation by intra-luminal administration of Poly I: C led to exacerbated enteropathy and villous atrophy. The histological changes following TLR3 agonism can be attributable to rapid IFN-β secretion, which may favor a characteristic Th1 profile in celiac disease [128]. Furthermore, TLR3 establishes a pro-inflammatory environment by promoting an isolated increase in TNF-α producing lymphoid cells and ceasing production of the anti-inflammatory cytokine IL-10 [129].

### Role of TLR3 in allergy

Similar to the pathogenesis of autoimmune diseases, the pro-inflammatory downstream modulators in the TLR3 pathway are also noteworthy in allergic inflammation in the respiratory tract and skin. In vivo trials highlight the role of TLR3 in upper airway allergic diseases, demonstrating that seasonal allergic rhinitis exacerbated by pollen exposure increases TLR3 mRNA expression and immunoreactivity in the nasal mucosa [130]. This upregulation of TLR3 during pollen exposure is believed to contribute to airway inflammation, as evidenced by heightened production of antiviral proteins following dsRNA challenge in allergic rhinitis patients during pollen season [131]. Concurrent stimulation with TLR3 and pollen increases production of IFN-β and IL-32 in nasal mucosa. Past studies have demonstrated that nasal epithelial cell cultures exhibit TLR3-dependent upregulation of IFN-β and IL-6, which can be induced by IL-32 [132, 133]. These findings support the significance of TLR3 in inducing cytokine-dependent upper airway inflammation in allergic rhinitis.

TLR3 may also aggravate asthma [134]. The pro-inflammatory state induced by TLR3 in the presence of allergic stimulants in mice contributes to increased dendritic cell migration to the lungs, T-cell proliferation, and expression of T-cell specific cytokine RNA [135]. Furthermore, airway smooth muscle cells react to TLR3 activation by upregulating chemokine synthesis, potentially contributing to airway obstruction and exacerbation of asthmatic symptoms [136]. TLR3 activation of both the innate and adaptive immune systems in the airway is associated with the amplification of the inflammatory cascade characteristic of asthma.

Atopic and contact dermatitis, which are inflammatory skin diseases rooted in hypersensitivity reactions, are closely regulated by TLR3 activation of the inflammatory response. TLR3 enhances allergic inflammation in mice by promoting leukocyte infiltration and edema upon skin irritation in contact dermatitis [137]. The heightened hypersensitivity reaction may be linked to elevated local cytokines and chemokines in wild-type mice compared to TLR3-deficient mice. Similar findings are observed in atopic dermatitis in mice [138]. The absence of TLR3 not only alleviates skin inflammation in allergic diseases, but also reduces the itch reflex in dry-skin-induced conditions [139]. Clinically, increased TLR3 expression in the stratum corneum in atopic dermatitis patients is correlated with increased disease severity, which may be attributable to TLR3-induced production of type 2 cytokines that drive eosinophil recruitment [140]. The pathogenic role of TLR3 in allergic skin diseases encompasses both inflammation at a molecular level and exacerbation of clinical symptoms, emphasizing the intricate interplay between immune response modulation and dermatological manifestations.

### Clinical implications of TLR3 agonists in non-malignant diseases

While cancer immunotherapy remains the primary focus of TLR3 agonist clinical use, the aforementioned small molecules have also shown promise in treating non-malignant diseases. Rintatolimod has been extensively studied as a therapy for chronic fatigue syndrome (CFS), a condition hypothesized to result from a combination of viral infection and genetic polymorphisms that impair pathogen clearance [141]. Known for its ability to upregulate NK cell functionality and reduce viral loads, Rintatolimod has been considered a tolerable drug that effectively improves the quality of life in CFS patients. Although Rintatolimod has yet to achieve FDA approval in the U.S., it has been approved for severe CFS in Argentina since 2016 and has been available through early access programs in Europe and Canada since 2015. Despite a failed clinical trial for HIV patients [142], where Rintatolimod did not prevent declines in CD4 counts, it was successful in enhancing the IgA response in healthy adults receiving the influenza vaccine, demonstrating its potential as an adjuvant in antiviral prevention [143]. Additionally, ongoing trials are evaluating Rintatolimod as a therapy for COVID-19 in cancer patients (NCT04379518) and post-COVID fatigue (NCT05592418), further expanding the clinical applications of this TLR3 agonist.

Conversely, Poly ICLC has shown limited success beyond its anti-cancer applications. A clinical trial in HIV patients showed that while Poly ICLC was well-tolerated and upregulated IFN signaling, it did not significantly affect CD4 + T cell count or viral load [144]. As ongoing trials continue to monitor the clinical efficacy of Poly ICLC in HIV patients (NCT01127464, NCT06665646), there has yet to be compelling evidence supporting its broader therapeutic benefits outside of cancer.

Beyond cancer and viral infections, TLR3-based therapies have not progressed to clinical trials for other diseases, such as autoimmune disorders or allergies. Nevertheless, several antagonists of other TLRs are being evaluated in ongoing clinical trials for autoimmune diseases. For example, a TLR4 monoclonal antibody inhibitor showed no improvement in RA [145]. Meanwhile, several trials of TLR7 and TLR9 antagonists are underway for SLE and psoriasis, offering hope for more effective treatments in these diseases [146]. While there are no clinical trials involving TLR3 antagonists, an anti-CXCL10 antibody has shown efficacy in combination with methotrexate for RA [147]. Since CXCL10 is regulated by TLR3 and is suggested to mediate TLR3-related inflammatory responses in RA [148], there is potential for the development of TLR3 antagonists as therapeutic options for autoimmune diseases in the future.

## Conclusions

TLR3 plays a crucial role in modulating both innate and adaptive immunity and has been shown to be a critical regulator in cancer, infectious diseases, autoimmune disorders, and allergy. High TLR3 expression does not guarantee an improved prognosis, as both beneficial and pathological roles have been suggested in human immunopathology. This dual nature of TLR3, being protective in some contexts and pathological in others, underscores its role as a double-edged sword in immunopathology. TLR3 activation enhances antiviral defenses and promotes pathogen clearance. However, excessive or dysregulated signaling can exacerbate chronic inflammation, tissue damage, and disease progression.

In cancer, TLR3 exhibits a dichotomous role, with its expression correlating with both good and poor prognoses depending on tumor subtype and microenvironment. Nevertheless, the pro-inflammatory nature of the TLR3 pathway laid the foundation for the development of TLR3 agonists in cancer immunotherapy. Noteworthy examples include Poly I: C derivatives like Poly A: U, rintatolimod, and Poly ICLC, which are under evaluation in clinical trials for their ability to enhance anti-tumor immune responses. Poly A: U demonstrated significant survival benefits in gastric cancer patients when combined with chemotherapy. Rintatolimod has shown promise as an immune adjuvant, enhancing dendritic cell maturation and type I IFN production. Poly ICLC, a more stable and potent derivative of Poly I: C, has demonstrated efficacy in inducing robust immune responses in head and neck squamous cell carcinoma and melanoma, where it activates local and systemic immunity and synergizes with immune checkpoint inhibitors to amplify anti-tumor effects.

Beyond oncology, TLR3 plays a significant role in the pathogenesis of autoimmune diseases and allergies, where its activation leads to amplified cytokine production and immune cell recruitment, contributing to the exacerbation of symptoms. Upregulation of IFN synthesis following TLR3 activation in these diseases often results in reduced clearance of inflammatory debris and increased infiltration of leukocytes into resident tissue. Targeting the TLR3 signaling pathway to alleviate tissue damage and chronic inflammation may offer a promising therapeutic approach to mitigate disease progression.

Recent clinical trials have explored TLR3 agonists in various diseases, but limited success highlights the need for precise modulation to balance therapeutic benefits with adverse effects. Emerging applications, like rintatolimod for chronic fatigue syndrome and post-COVID fatigue, demonstrate the potential of TLR3-targeted strategies. Future research should focus on context-specific effects of TLR3 activation and the development of targeted, safer therapies to harness its dual role for improved clinical outcomes.

## Data Availability

No datasets were generated or analysed during the current study.

## Abbreviations

AP1: Activator Protein 1
CD: Cluster Of Differentiation
CSC: Cancer Stem Cells
CVB: Coxsackievirus B
DC: Dendritic Cell
DNA: Deoxyribonucleic Acid
dsRNA: Double-Stranded Ribonucleic Acid
EMCV: Encephalomyocarditis Virus
GM-CSF: Granulocyte-Macrophage Colony-Stimulating Factor
HBV: Hepatitis B Virus
HER-2/neu: Human Epidermal Growth Factor Receptor 2
hPIV-1: Human Parainfluenza Virus 1
HSV: Herpes Simplex Virus
IFN: Interferon
IL: Interleukin
IRF-3: Interferon Regulatory Factor 3
M1: Macrophage-1 antigen
mRNA: Messenger Ribonucleic Acid
NF-κB: Nuclear Factor Kappa B
NSCLC: Non-Small Cell Lung Carcinoma
NCT: National Clinical Trial
ORR: Objective Response Rate
OS: Overall Survival
PAMP: Pathogen Associated Molecular Pattern
PFS: Progression Free Survival
